# Spatial and Temporal Variability of Rainfall in Eastern Amazon during the Rainy Season

**DOI:** 10.1155/2015/209783

**Published:** 2015-02-22

**Authors:** Douglas Batista da Silva Ferreira, Everaldo Barreiros de Souza, Bergson Cavalcanti de Moraes, Luiz Gylvan Meira Filho

**Affiliations:** ^1^Vale Institute of Technology, Boaventura da Silva Street 955, 66055-090 Belém, PA, Brazil; ^2^Post-Graduate Program in Environmental Sciences, Federal University of Pará, Augusto Corrêa Street 1, 66075-110 Belém, PA, Brazil

## Abstract

Empirical orthogonal functions (EOF) and composites analysis were employed on pentad data in order to investigate the tropical atmospheric-ocean patterns over the Atlantic Ocean and the spatial-temporal characteristics of the rainfall in eastern Amazon during the peak of the rainy season (February to April). The EOF results evidenced that the Intertropical Convergence Zone (ITCZ) is the main rainfall-producing system in eastern Amazon during the rainy season. Conditions associated with the southward SST gradient in the intertropical Atlantic formed the dynamic patterns that favored the position of the ITCZ to south of the equator, thus explaining the predominance of positive precipitation anomalies in eastern Amazon, especially in the state of Maranhão and northeastern Pará during the February and April months.

## 1. Introduction

**Figure 1:**
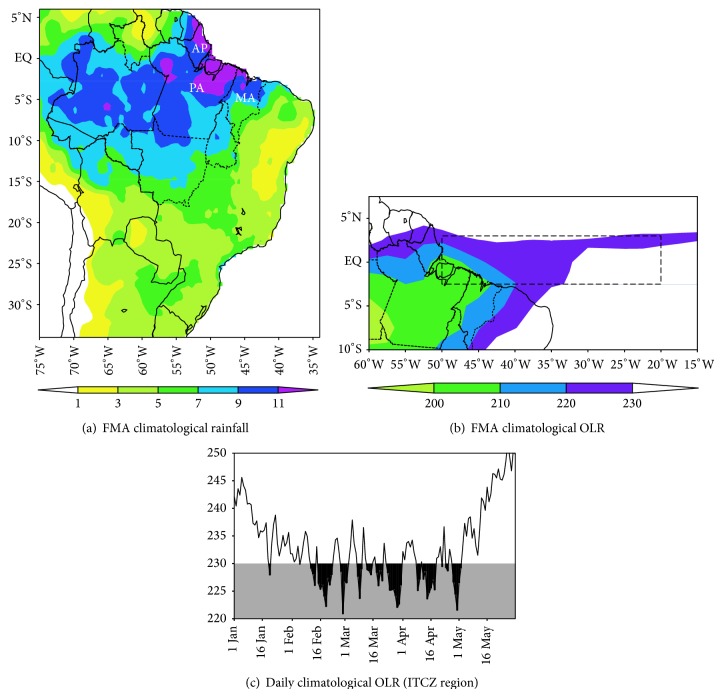
Climatology (1981/2010) for the FMA period of the (a) rainfall (mm/day) in South America and (b) OLR (W/m^2^) in the Atlantic Ocean and eastern Amazon. (c) Time evolution from January to May of the daily climatological OLR (W/m^2^) averaged in ITCZ region (2.5S/2.5N and 40W/10W; see location in dashed box). Black dashed lines in (a) show the limits of the states of the Brazilian Amazon, emphasizing AP (Amapá), PA (Pará), and MA (Maranhão) in eastern Amazon.

The Amazon in South America and equatorial Africa and Indonesia form the three main centers of action of deep convection around the global tropical belt [1]. In particular, the Amazon exhibits a high spatial and temporal variability of rainfall with a very pronounced seasonality, such that the rainy and dry seasons typically occur during the months of summer/autumn and winter/spring, respectively [2]. The focus of the present paper is on the spatial distribution of rainfall in eastern Amazon (see location in Figure 1(a)) during the period in which the maximum (the peak of the rainy season) of the annual cycle is reached, that is, February to April (FMA). The climatological mean (1981–2010) for FMA (Figure 1(a)) clearly shows that the continental precipitation maximum is concentrated over the eastern Amazon, with values above 11 mm/day in most of the states of AP (Amapá), PA (Pará), and MA (Maranhão).

Previous observational studies have investigated the climatological rainfall patterns in the Amazon [3] and reported that the pluviometric variability is explained by the manifestation of a wide range of meteorological systems, like the Intertropical Convergence Zone (ITCZ), squall lines, influences of frontal systems, South Atlantic Convergence Zone (SACZ), and meso- and local-scale systems [4, 5].

On the other hand, the Amazonian climate variability is influenced by the large-scale tropical climatic modes associated with the El Niño/Southern Oscillation (ENSO) cycle in the Pacific Ocean [6, 7] and with the interhemispheric sea surface temperature (SST) gradient phases in the Atlantic Ocean [8, 9]. Of relevance to the scope of the present work is the Atlantic gradient that appears dominant during the austral autumn. Typically, a northward (southward) gradient features simultaneous positive/negative (negative/positive) SST anomalies in the tropical north/south basin of the Atlantic Ocean [8]. Such meridional gradients hydrostatically control the sea level pressure, wind patterns, and the moisture convergence in the lower-levels, thereby influencing the latitudinal positioning of the ITCZ during its seasonal migration towards the south Atlantic [10].

The configuration of the deep tropical convection, as depicted by the climatological OLR in Figure 1(b), reveals the presence of a zonal band containing values less 230 W/m^2^ which is related primarily to the ITCZ between the equatorial Atlantic and eastern Amazon. The daily climatology of the OLR averaged in the ITCZ region (see box in Figure 1(b)) shows that the period with most intense convection occurs during the months of February to April (black contours in Figure 1(c)), confirming the rainy season in these months. Thus, objective of this study is to investigate the ocean-atmosphere patterns over the tropical domain covering the Atlantic Ocean and South America with emphasis on the identification of the characteristics associated with the ITCZ and its impacts on the spatial distribution of rainfall in eastern Amazon.

## 2. Data and Methods

The latest version of daily rainfall data from the Climate Prediction Center (CPC)/National Centers for Environmental Prediction (NCEP) [11] is used to investigate the spatial pluviometric variability in eastern Amazon. These data are derived from the observation network of surface meteorological stations in Brazil on a resolution of 0.5°/0.5° latitude/longitude. Silva et al. [12] described the gauge-only rainfall data quality control and analysis system which has been applied to develop these improved gridded daily rainfall analyses over Brazil. In addition, they reported that the use of such information could be applied in validation of numerical models and real-time regional monitoring and climate studies. In addition, we used zonal and meridional wind data generated by the reanalysis system of NCEP/National Center for Atmospheric Research (NCAR) [13], outgoing longwave radiation (OLR) data derived from National Oceanic and Atmospheric Administration (NOAA) polar-orbiting meteorological satellites [14], and sea surface temperature (SST) data obtained from NCEP [15]. All these observational data were temporally interpolated into pentads, that is, five-day means for the months of February to April during 1981 to 2010. For February, the last pentad includes only 26, 27, and 28 while in March it covers the last six days. The pentad anomalies were calculated taking into account the long-term mean (30 years: 1981–2010) of the respective periods.

The statistical technique named as Empirical Orthogonal Function (EOF) is used in order to determine the ITCZ as the dominant mode of climate variability in the tropical Atlantic/eastern Amazon during the rainy season. The mathematical formulation of the EOF is fully described by Kutzbach [16] and the methodology employed in the present work is similar to approach taken by De Souza et al. 2005 which applied EOF to investigate the climate variability in tropical Brazil. Thus, EOF analysis was performed on gridded pentad OLR anomalies for February to April months during the 1983 to 2012 period, totaling 30 rainy seasons, over the domain comprised between 15°N to 15°S and 60°W to 0° which is the region of the Atlantic ITCZ. The annual cycle was removed with the calculation of pentad OLR anomalies before applying the EOF. The EOF computation is based on the correlation matrix and the eigenvectors for each mode are presented as correlation patterns, so that the signals correspond to the OLR anomalies; that is, negative/positive values are interpreted as anomalously enhanced/weakened convection. Each season was considered as an independent event, and the statistical significance was assessed using Student's *t*-test at a confidence level of 95%. Furthermore, the method of North [17] was used to verify the significance of EOF physical modes. The time series of the principal component (PC) of the EOF dominant modes are used to select significant OLR events that are defined when PC coefficient exceeds the threshold of +1 standard deviation (or −1 depending of the EOF signal). These events are used for the calculation of the composites in order to investigate the configuration of OLR patterns and the spatial distribution of the rainfall in eastern Amazon. These composites for OLR, wind, SST, and rainfall patterns are analyzed with emphasis on the anomalies statistically significant at the confidence level of 95% according to the *t*-test as in [9].

## 3. Results

### 3.1. EOF Analysis

**Figure 2:**
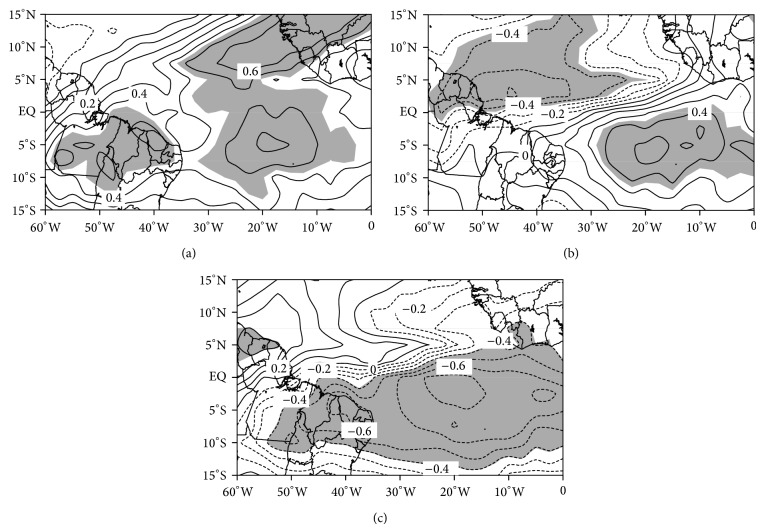
Spatial pattern of (a) EOF1 for February, (b) EOF2 for March, and (c) EOF1 for April performed on pentad OLR anomalies (1983–2012).

Figure 2 illustrates the dominant modes of OLR variability for February, March, and April with an explained variance of 16.5%, 9.2%, and 16.1%, respectively. All modes are statistically independent of the other modes according to North [17] criterion. Contours shaded in gray represent eigenvectors at a confidence level of 95% by Student's *t*-test. The spatial patterns obtained from the first mode (EOF1) for February (Figure 2(a)), second mode (EOF2) for March (Figure 2(b)), and first mode (EOF1) for April (Figure 2(c)) resemble the well-known ITCZ-related OLR band along the equatorial Atlantic and eastern Amazon. For February and April the respective significant positive and negative OLR areas show the ITCZ band influencing eastern Amazon and equatorial Atlantic, while in March the positive OLR pattern is over the south Atlantic.

**Figure 3:**
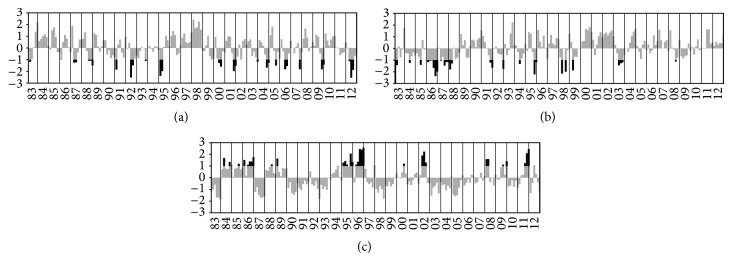
Time series of the PC coefficients associated with (a) EOF1 February, (b) EOF2 March, and (c) EOF1 April. The PCs in black with threshold below −1 standard deviation for February and March and above +1 standard deviation for April represent ITCZ events selected for each pentad.

Figure 3 shows the PC time series corresponding to the EOF1 for February, EOF2 for March, and EOF1 for April, in which high temporal variability of the EOF patterns during 1981 to 2010 period is observed. Black bars in Figure 3 show significant PC values. The area with positive eigenvectors in February and March with significant negative PC (less than −1 standard deviation) represents events OLR associated with intensified ITCZ convective activity, while in April the negative eigenvectors with significant positive PC (greater than +1 standard deviation) represent the same events. Thus, it was possible to select objectively the events associated with the ITCZ in each month of the years studied.  Table 1 enumerates the pentads in each month selected by the criteria abovementioned with total of 29 events for February and also in March and 35 events for April. These events will be used in the composites analyses to follow.

### 3.2. Composites

#### 3.2.1. February

**Figure 4:**
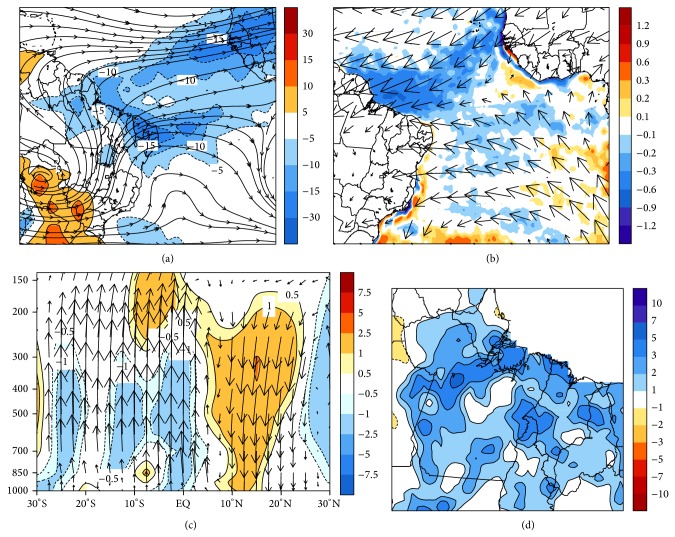
Composites for February (a) OLR anomalies in (W/m^2^) and wind at 200 hPa (stream lines), (b) SST anomalies (shaded in °C) and wind at 925 hPa (vectors m/s), (c) tropospheric circulation of Hadley in 050°W and Omega anomalies in colors, and (d) rainfall anomalies (mm/pentad), corresponding to the objectively selected events in the EOF1.

In Figure 4(a), the composites of the OLR anomalies indicate two large areas zonally oriented, with values lower than −10 W/m^2^. The first can be seen around the 05°N latitude and the second positioned over Northeast Brazil (NEB). This pattern suggests the formation of a double ITCZ band, which is normally associated with the rainy years. The double ITCZ usually happens to south of the main convective band in the first months of the year (FMA) with few-day duration [18]. The streamlines at 200 hPa wind characterize well the end of summer in the Southern Hemisphere, when the Bolivian High (BH) is well defined at approximately 15°S and 063°W, inducing tropical convection in eastern Amazon [19].

In Figure 4(b), the spatial distribution of SST anomalies shows a region with negative values in the subtropical North Atlantic (blue colors). Although the southern basin does not present a well-defined heating pattern, there is a presence of an interhemispheric thermal gradient and the band of cloudiness tends to arrange in latitudes where there is a change of signal SST anomalies [9]. This typical condition means that the ITCZ convection band tends to organize in the southern latitudes; that is, total rainfall above the climatology is expected in eastern Amazon [20]. In 925 hPa level the convergence of the trade winds is present close to equator, favoring the formation of convective cloudiness in this region. With respect to vertical movement, Figure 4(c) shows that in the northern latitudes there is downward movement, while in the southern latitudes there is upward movement of the air. This tropospheric configuration is directly associated with presence of the ITCZ over the equatorial Atlantic Ocean, according to Figures 4(a) and 4(b), formed with double band and, thus, coinciding with the two areas containing negative Omega anomalies (equator and 10°S), where tropical convection is observed.

The result of these large-scale conditions is shown in Figure 4(d), whose spatial distribution of rainfall anomalies presents positive values, especially near coastal region, where the ITCZ with double band exerts a greater impact due to oceanic influence. Thus, Pará and Maranhão states are those ones with higher values of pentad rainfall anomalies, with peaks of 3–5 mm/pentad at 05°S and 048°W, and in the coastal portion at 02°S and 047°W, and also 5–7 mm/pentad in the lower Amazon river (03°S and 055°W).

#### 3.2.2. March

**Figure 5:**
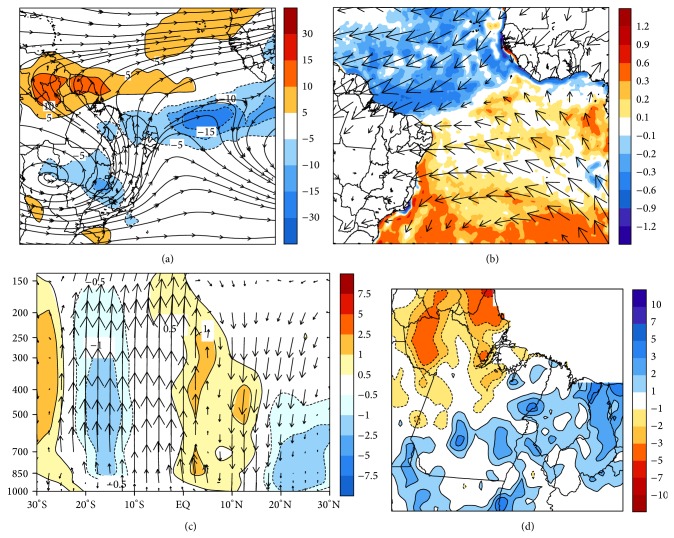
As in Figure 4, but for March composites, corresponding to the objectively selected events in the EOF2.

Over the Atlantic Ocean, Figure 5(a) shows a pattern of negative OLR anomalies below equator line. Thus, the equatorial trough tends to be positioned, generally, at latitudes in the Southern Hemisphere. March marks the beginning of austral autumn. However, the circulation of high levels shows summer characteristics, due to BH presence, denoted by anticyclonic circulation (between Bolivia and the Mato Grosso and Rondônia states in Brazil) which is also associated with the trough over NEB. Another factor that strongly contributes to the displacement of large-scale systems is interhemispheric thermal gradient, which can be seen in Figure 5(b). The opposite pattern with negative SST anomalies in the Northern Hemisphere (blue colors) and positive in Southern Hemisphere (colors yellow to red) relates to the years in which ITCZ reaches its lowest latitudes. As for the horizontal wind at 925 hPa, the confluence of the trade winds occurs approximately at 05°S latitude, coinciding with the OLR anomalies and SST.

The tropospheric circulation related to the Hadley (Figure 5(c)) clearly illustrated that in latitudes that comprise the eastern Amazon, the upward movement is favored, since the lower layers (850 hPa) to the highest (150 hPa), especially between 20°S and 05°S, are characterized by negative Omega anomalies. This means that the air converges near the surface and diverges around the tropopause. This pattern is related to the upward movement of air over the eastern Amazon. With the positioning of the ITCZ further southward, the positive rainfall anomalies tend to be restricted to the NEB (Figure 5(d)). On the other hand, as we move to north along the coast, the anomalies change the sign. Thus, Amapá and parts of the lower Amazon river and Marajó island present deficit rainfall coinciding with the positive OLR anomalies. In contrast, the southeastern Pará, Maranhão, and most of northern Tocantins present anomalously increased rainfall.

#### 3.2.3. April

**Figure 6:**
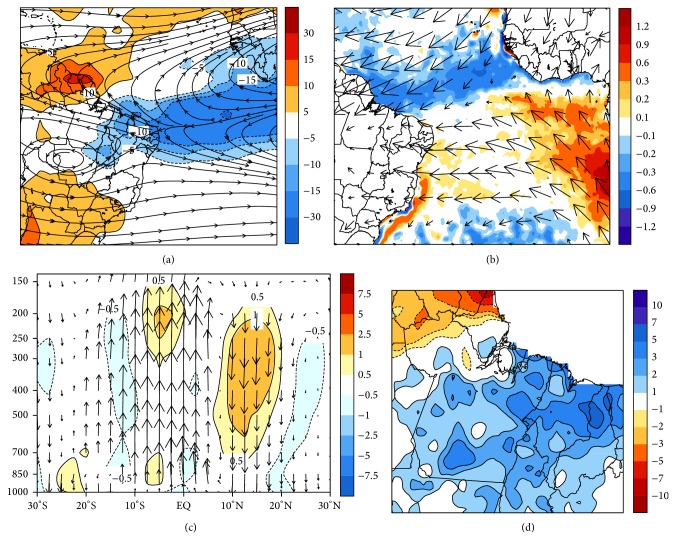
As in Figure 4, but for April composites, corresponding to the objectively selected events in the EOF1.

The OLR anomalies and the streamlines at 200 hPa are shown in Figure 6(a). It is observed that BH appears further northward compared to the previous months composites (anticyclonic circulation over Mato Grosso in 15°S and 058°W) and a diffluence wind at 05°S latitude, connected to the ITCZ movement in high levels. The OLR pattern shows an area of deep convection in the subtropical Atlantic, containing negative anomalies ranging between −15 W/m^2^ and −30 W/m^2^ (blue colors). Associated with these dynamic conditions, the trend is that there is greater amount of convective clouds; consequently, most of the significant volumes of rainfall are observed in eastern Amazon. Looking at Figure 6(b), the 925 hPa wind, a convergence region can be seen between 00° and 05°S latitudes. Regarding the composite of the SST anomalies, it is verified negative values over the subtropical North Atlantic, which were responsible for intensifying the northeast trade winds and, thus, move the ITCZ band to the Southern Hemisphere. On the other hand, the positive anomalies positioned over the south subtropical Atlantic weaken the southeastern trade winds.

In terms of the Hadley circulation (Figure 6(c)), despite the lower magnitude of the Omega anomalies, the vectors indicate a predominance of convection between the equator line and 10°S. This upward motion of air corresponds to the region with negative OLR anomalies and comprises the area where there is a confluence of the trade winds; that is, all these characteristics are related to the periods of ITCZ with intense activity over the South Subtropical Atlantic during April.

The combined action of these factors can be seen in the composites of rainfall anomalies observed in Figure 6(d), with a spatial pattern showing an excess rainfall across eastern Amazon, especially in the triple border among the states of Pará, Maranhão, and Tocantins. Also the coastal region of Pará and Maranhão showed significant rainfall anomalies, above 3 mm/pentad. In northern Amapá, the anomalies are negative due to the ITCZ positioning in southern latitudes, such as in April in rainy years. Thus, the dynamic structure of the ocean-atmosphere patterns related to this wet condition in eastern Amazon clearly resembles the ITCZ activity in the Atlantic and the presence of this weather system over the subtropical Atlantic Ocean is responsible for anomalously rainy periods.

## 4. Conclusions

The EOF analysis employed on pentad OLR anomalies evidenced that the ITCZ is the main mode of climate variability over the intertropical Atlantic Ocean and it is the main rainfall-producing system in eastern Amazon during the rainy season.

The composite analysis revealed a robust dynamical pattern related to the ITCZ in the south equatorial Atlantic, with the presence of Atlantic southward SST gradient that in turn contributes directly to the abundant rainy season in eastern Amazon, so that the positive rainfall anomalies are observed especially in the Maranhão state and northeastern Pará. These significant events of rainfall in general are associated with the pronounced elevation of the Amazon rivers, flooding in several municipalities, among others conditions that impact directly the local society. Therefore, this study contributed to a better understanding about the rainfall effects related to the ITCZ during the rainy season in eastern Amazon.

## Figures and Tables

**Table 1:** Dates of pentads events selected objectively by PC obtained by the EOF technique, applied in February, March, and April (1983–2012), separately.

Year	Events—February (1st mode)	Events—March (2nd mode)	Events—April (1st mode)
1983	06—10	01—05, 06—10	06—10
1984	—	11—15	21—25, 26—30
1985	—	11—15	16—20
1986	01—05	01—05, 06—10, 11—15, 16—20, 21—25, 26—31	01—05, 11—15, 16—20, 21—25
1987	06—10, 11—15	06—10, 16—20, 21—25, 26—31	—
1988	16—20, 21—25, 26—28	01—05, 06—10	16—20
1989	—	—	01—05
1990	—	—	—
1991	01—05	21—25, 26—31	—
1992	11—15, 16—20	26—31	—
1993	21—25	—	—
1994	—	11—15	16—20
1995	01—05, 06—10	21—25, 26—31	01—05, 06—10, 11—15, 21—25, 26—30
1996	—	—	06—10, 11—15, 16—20, 21—25, 26—30
1997	—	—	26—30
1998	—	06—10, 16—20	—
1999	—	06—10	—
2000	11—15, 16—20	—	16—20
2001	21—25, 26—28	—	—
2002	—	—	06—10, 11—15, 16—20
2003	26—28	11—15, 16—20, 21—25	—
2004	21—25, 26—28	—	—
2005	16—20	—	—
2006	11—15, 16—20	—	—
2007	21—25	—	—
2008	—	16—20	01—05, 06—10
2009	21—25, 26—28	—	16—20, 26—30
2010	—	—	—
2011	—	—	16—20, 21—25, 26—30
2012	06—10, 11—15, 16—20	—	11—15

Total	29 events	29 events	35 events
